# Multiplexed RT-qPCR Coupled with Whole-Genome Sequencing to Monitor a SARS-CoV-2 Omicron Variant of Concern in a Hospital Laboratory Setting in Latvia

**DOI:** 10.3390/diagnostics13223467

**Published:** 2023-11-17

**Authors:** Baiba Niedre-Otomere, Inara Kampenusa, Julija Trofimova, Jevgenijs Bodrenko, Reinis Vangravs, Girts Skenders, Sergejs Nikisins, Oksana Savicka

**Affiliations:** National Microbiology Reference Laboratory of Latvia, Laboratory “Latvian Centre of Infectious Diseases”, Laboratory Service, Riga East University Hospital, Linezera Street 3, LV-1006 Riga, Latvia; inara.kampenusa@aslimnica.lv (I.K.);

**Keywords:** multiplexed RT-qPCR, whole-genome sequencing, SARS-CoV-2 Omicron variant of concern, SARS-CoV-2 signature mutations: ΔH69/V70, E484A, N501Y

## Abstract

At the end of 2021, the SARS-CoV-2 Omicron variant of concern (VOC) displaced the previously dominant Delta VOC and enhanced diagnostic and therapeutic challenges worldwide. Respiratory specimens submitted to the Riga East University Hospital Laboratory Service by the central and regional hospitals of Latvia from January to March 2022 that were positive for SARS-CoV-2 RNA were tested by commercial multiplexed RT-qPCR targeting three of the Omicron VOC signature mutations: ΔH69/V70, E484A, and N501Y. Of the specimens tested and analyzed in parallel by whole-genome sequencing (WGS), 964 passed the internal quality criteria (genome coverage ≥90%, read depth ≥400×) and the Nextstrain’s quality threshold for “good”. We validated the detection accuracy of RT-qPCR for each target individually by using WGS as a control. The results were concordant with both approaches for 938 specimens, with the correct classification rate exceeding 96% for each target (CI 95%); however, the presumptive WHO label was misassigned for 21 specimens. The RT-qPCR genotyping provided an acceptable means to pre-monitor the prevalence of the two presumptive Omicron VOC sublineages, BA.1 and BA.2.

## 1. Introduction

The increasing viral fitness of the sequentially dominating SARS-CoV-2 variants and subvariants has been driving the path of the COVID-19 pandemic [1]. Therefore, diagnostic laboratories, academics, and commercial developers of diagnostic reverse-transcription polymerase chain reaction (RT-PCR) SARS-CoV-2 tests and those for variants of concern (VOCs) identification were faced with continual challenges [2,3,4,5,6,7]. Several approaches enabled SARS-CoV-2 genotyping and VOC detection [8,9]. The Alpha VOC (B.1.1.7) showed *S* gene target failure with several RT-PCR tests for SARS-CoV-2 RNA due to the deletion of two adjacent codons for H69 and V70 in the N-terminal domain. This signature transformed into an indicative test for the Alpha VOC [10] and later for the Omicron VOC (B.1.1.529, BA.1, BA.4, and BA.5) [11,12] as it resurfaced and remained in several of its sublineages. Allele-specific quantitative RT-PCR assays (RT-qPCR) target the signature mutations of VOCs, which present as single nucleotide polymorphisms and insertion-deletion polymorphisms. RT-qPCR has already been successfully used to detect and monitor the de-escalated VOCs [13,14,15], with the simultaneous detection of the targeted signature mutations in a short turnaround time being the main advantage. Targeting multiple S protein substitutions by combining two or more commercial test kits [16,17] or extensive panels of single nucleotide polymorphism (SNP) targets [5,18] with the inclusion of variant-independent targets [5] enabled identification of variants. During each transition phase from one VOC to the next, the discriminatory capacity of the VOC assay designated for the circulating variant could be increased by the inclusion of targets from the preceding VOC [5,19,20].

The advent of an antigenically distinct Omicron VOC was a landmark due to its unprecedented capacity to escape neutralization by not only infection, vaccine, or hybrid-induced antibodies but also by most monoclonal therapeutic antibodies [21,22,23,24]. The immune evasion properties dramatically narrowed down the spectrum of neutralizing antibodies for the treatment of patients with certain medical conditions with a risk for advancement to severe COVID-19 [25]. The viral fitness advantage of the Omicron VOC was conferred by a particularly striking number of amino acid substitutions in the main targets of neutralizing antibodies [26,27] within the virus S protein [28], namely, the receptor binding (RBD) and N-terminal domains.

The first Omicron VOC (BA.1) was detected by whole-genome sequencing (WGS) in Latvia by the National Microbiology Reference Laboratory (NMRL) from a respiratory specimen submitted on December 8, 2021—twelve days after the World Health Organization (WHO) declared it a VOC [29]. Within a few weeks, the sublineages BA.1 and BA.1.1 constituted the majority (57%) of all sequenced SARS-CoV-2 genomes submitted on December 27 at the NMRL, while by the second week of January 2022, the Omicron VOC had already reached 90% of sequenced genomes. To respond to the increase in the Omicron VOC in the Delta-dominated SARS-CoV-2 variant landscape in Latvia, NMRL introduced the commercial multiplexed RT-qPCR assay targeting a few of the Omicron VOC signature mutations in the *S* gene: a deletion in the N-terminal domain (ΔH69/V70) and two SNPs in the RBD (E484A and N501Y). The N501Y substitution arose independently in the second half of 2020 by natural selection pushed by host immunity [30] within three geographically distant lineages, B.1.1.7, B.1.351, and P.1, corresponding to the WHO labels Alpha, Beta, and Gamma VOCs. This signature has been maintained across all of the VOCs, except Delta [31]. E484A first appeared in the Omicron VOC, replacing the E484K from the previous Alpha (B.1.1.7) [32], Beta (B.1.351), and Gamma (P.1) VOC [33]. ΔH69/V70 emerged in the Alpha VOC only to reappear again in the Omicron VOC [1].

The ECDC recommends continuous verification of RT-PCR-based VOC assays via genomic approaches [34]. This study validated the performance of the multiplexed RT-qPCR assay for the Omicron VOC via WGS. Respiratory specimens submitted to our laboratory from January 3 to March 30 2022 and positive for SARS-CoV-2 RNA were included.

## 2. Materials and Methods

### 2.1. Setting

The Riga East University Hospital (REUH) Laboratory Service known as the “Latvian Centre of Infectious Diseases” provides diagnostic services for central and regional hospitals, health centers, and general practitioners. Of the 4030 SARS-CoV-2-positive respiratory specimens tested with the multiplexed RT-qPCR VOC test, 76% (*n* = 3070) were submitted from the inpatient and outpatient departments of the REUH, 22% (*n* = 905) from general practitioners’ clinics and health centers, and 1% (*n* = 55) from nine other hospitals.

### 2.2. Detection of SARS-CoV-2 RNA

We extracted SARS-CoV-2 RNA using the NUCLISENS easyMAG system V.2.0 (Biomérieux, Marcy-l’Étoile, France), reversely transcribed it, and amplified the cDNA by several alternative assays: the Alinity m SARS-CoV-2 AMP KIT (Abbott, Abbott Park, IL, USA) detects the *RdRp* and *N* genes, the Allplex SARS-CoV-2 Master Assay (Seegene, Seoul, Republic of Korea), which determines the *RdRp*, *S*, and *N* genes and the *E* gene of all sarbecoviruses, the RealStar SARS-CoV-2 RT-PCR Kit 1.0 RUO (Altona Diagnostics, Hamburg, Germany), targeting *S* and *E* genes, the RealAccurate Quadruplex SARS-CoV-2 PCR Kit (Patho Finder, Maastricht, The Netherlands) with the detection of *RdRp* and *N* genes, Xpert Xpress CoV-2 (Cepheid, Sunnyvale, CA, USA) detects *E* and *N2* genes, the Aptima SARS-CoV-2 assay Panther System V.2.2.0.4 (Hologic, Marlborough, MA, USA), targeting *Orf1ab*, and an automated system for SARS-CoV-2 RNA extraction, reverse transcription, and the amplification of cDNA Cobas 6800 (Roche, Basel, Switzerland) targeting *E* and *Orf1a*.

### 2.3. Detection of SARS-CoV-2 Variants by Multiplexed RT-qPCR

From SARS-CoV-2-positive respiratory specimens, RNA was extracted with an automated workstation, Nimbus (Seegene). The RNA was subjected to multiplexed one-step RT-qPCR with the Novaplex SARS-CoV-2 Variants VII Assay (Seegene) employing the CFX96 Touch Real-Time PCR Detection System (Biorad, Hercules, CA, USA). The PCR conditions defined by the manufacturer were followed. The internal control of an unspecified endogenous gene was provided by the manufacturer. The results were analyzed by Seegene software vers. 3.29, displayed by Seegene Viewer, and reported as Ct values for each gene sequence encoding ΔH69/V70, E484A, N501Y, and *RdRp*. We interpreted the results of the multiplexed RT-qPCR test in line with the manufacturer’s manual (V.1.00 12/2021). The presumptive WHO labels—Omicron and Delta—were assigned accordingly to the detected presence or absence of the three targets. Specimens positive for E484A and N501Y were interpreted as the Omicron VOC lineage BA.2, whereas specimens with other detected combinations of the targets were not specified, and we labeled those as “Other”. Specimens positive for the *RdRp* gene and an internal control were selected for this study.

### 2.4. Whole-Genome Sequencing

RNA from all SARS-CoV-2 RNA-positive respiratory specimens with a Ct value ≤ 30 was extracted by the MGIEasy Nucleic Acid Extraction Kit (MGI-Tech, Shenzhen, China), and libraries were prepared with the Illumina COVIDSeq kit (Illumina, San Diego, CA, USA) using ARTIC V4 primers and sequenced on the Illumina NextSeq550Dx (Illumina). The raw sequence data were analyzed with an in-house workflow (ws) by aligning filtered reads to the Wuhan-Hu-1 reference genome (MN908947.3) using bwa 0.7.17-r1198-dirty mem and samtools 1.12. Variant calls and consensus sequences were generated with freeBayes v0.9.21, vcflib 1.0.2/vcffilter, and iVar 1.3.1. Lineages were determined by PANGOLIN V.4.1 (Phylogenetic Assignment of Named Global Outbreak Lineages). The SARS-CoV-2 genomes included in this study were uploaded to the GISAID (Global Initiative on Sharing All Influenza Data) database (ID numbers are provided in Supplementary File S1).

### 2.5. Statistical Analysis

The detection accuracy of the multiplexed RT-qPCR was analyzed against the WGS results by confusion matrix-derived performance measures at a confidence interval (CI) of 95% for ΔH69/V70, E484A, and N501Y individually.

## 3. Results

### 3.1. Detection of SARS-CoV-2 Variants with Multiplexed RT-qPCR

**Figure 1:**
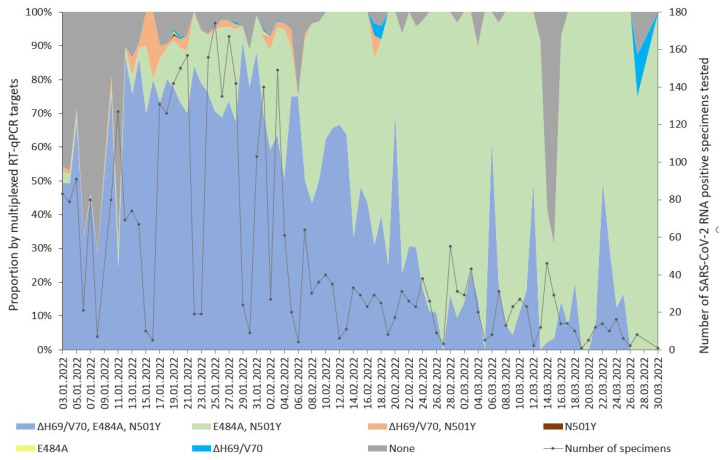
Presence of multiplexed RT-qPCR-targeted mutations within 3900 SARS-CoV-2 RNA-positive respiratory specimens tested by multiplexed RT-qPCR plotted against the submission date.

The average turnaround time of a multiplexed RT-qPCR test from submission of the respiratory specimen to a test result issued to a clinician was 8 h. In total, 3900 of the 4030 SARS-CoV-2 RNA-positive respiratory specimens analyzed by multiplexed RT-qPCR were positive for the *RdRp* gene and the internal control. We identified seven groups of SARS-CoV-2 RNA-positive specimens according to the presence of either all three targeted mutations (ΔH69/V70, E484A, and N501Y), either one of them, or combinations of two (ΔH69/V70 and N501Y or E484A and N501Y), as well as their absence (Figure 1).

The group with none of the targeted mutations detected and designated as the presumptive Delta VOC dominated during the first two weeks of January (Figure 1). The overall prevalence of the presumptive Delta VOC in January was 15.3% and ranged from 69% (87/127) of the SARS-CoV-2-positive specimens submitted on January 11 to 9% (6/67) on 14 January. The percentage of the detected presumptive Delta VOC dropped thereafter, only to reappear as a considerable percentage of tested SARS-CoV-2-positive specimens on 15 and 15 March, when it reached 59% (27/46) and 69% (20/29), respectively (Figure 1). In March, the prevalence of the presumptive Delta VOC was 11.9%. The ΔH69/V70 targeted by the multiplexed RT-qPCR test allowed for the simultaneous circulation of the two presumptive Omicron VOC sublineages BA.1 and BA.2. The peak of the Omicron VOC wave during the first five weeks of 2022 was led by the BA.1 sublineage (Figure 1), whereas from January 3, the proportion of BA.2 continued to rise (Figure 1), and on February 8, it exceeded 50% of the daily SARS-CoV-2-positive specimens tested. Until the end of March, the BA.2 sublineage completely displaced BA.1.

### 3.2. Whole-Genome Sequencing

**Figure 2:**
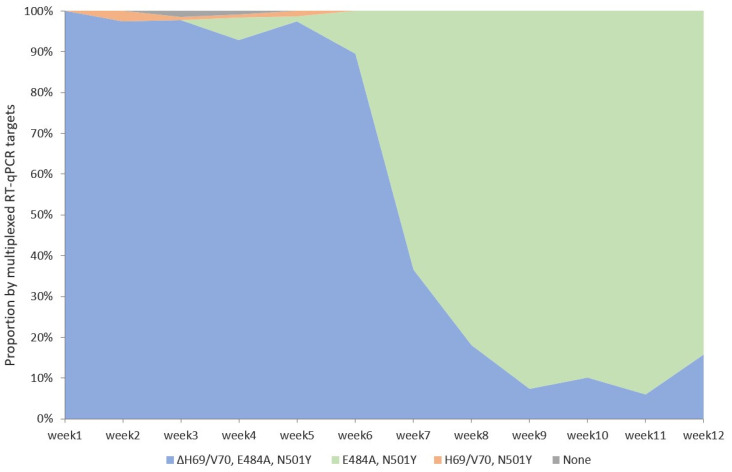
Presence of multiplexed RT-qPCR-targeted mutations in the genome sequences of 964 SARS-CoV-2 RNA-positive respiratory specimens plotted against the week of submission from January to March 2022.

In parallel with multiplexed RT-qPCR, the SARS-CoV-2 RNA-positive respiratory specimens with a Ct ≤ 30 were subjected to precision VOC detection by WGS. Of those, 964 SARS-CoV-2 genome sequences corresponded to internal quality criteria—genome coverage ≥90%, read-depth ≥400×, as well as Nextstrain’s benchmark for “good” [35]. The overall median average coverage was 3151.21 (min. 400.59, max. 5443.08), the median number of mapped reads was 852,245.50 (min. 87,222, max. 1,874,172), and the median reference genome coverage was 99.21% (min. 94.71%, max. 99.66%). The 964 sequenced SARS-CoV-2 genomes against the week of submission at the laboratory were plotted, and the presence of RT-qPCR-targeted mutations was depicted (Figure 2).

The Delta VOC was represented by three lineages: AY.121 (*n* = 1), AY.4 (*n* = 1), and B.1.617.2 (*n* = 2) from the specimens submitted in January, with a prevalence of 0.66%. There were no Delta VOCs detected in February or March (Figure 2).

The sublineages of the Omicron VOC completely covered the SARS-CoV-2 variant landscape since the first week of February (Figure 1). WGS analysis for the presence of mutations ΔH69/V70, E484A, and N501Y in the SARS-CoV-2 genomes confirmed the dynamics of both Omicron VOC lineages shown by genotyping tests, with an initial BA.1 dominance and subsequent decline accompanied by a simultaneous increase in BA.2 (Figure 2).

WGS analysis did not verify the appearance of the presumptive Delta VOC on March 14 and 15, as shown by multiplexed RT-qPCR (Figure 1). Of the 75 SARS-CoV-2-positive specimens tested by multiplexed RT-qPCR from the respective dates, none of the presumptive Delta VOCs qualified for WGS analysis. However, 18 other specimens from the respective dates were concordant by both methods for VOC detection and were classified as the Omicron VOC, PANGO lineages BA.1.1 (*n* = 1), BA.2 (*n* = 14), BA.2.22 (*n* = 1), and BA.2.9 (*n* = 2).

### 3.3. Validation of the Detection of the Three Targeted Omicron VOC Mutations ΔH69/V70, E484A, and N501Y) by Multiplexed RT-qPCR against WGS

The majority (*n* = 938) of the 964 multiplexed RT-qPCR specimens were recognized as concordant (Table S1) with the WGS results in terms of the presence and absence of the three targeted Omicron VOC mutations, whereas 2.7% (26/964) were identified as discordant (Table 1).

Importantly, the validation revealed that in 21 cases, the presumptive WHO label was assigned incorrectly (*n* = 20) or not assigned (*n* = 1; Table 1) by a multiplexed RT-qPCR test. Eight of the misassigned specimens were submitted on January 7 during the putative peak of the departing presumptive Delta VOC as presumed by multiplexed RT-qPCR (Figure 1). Moreover, of the 80 specimens tested on the respective date by VOC assay, only 20 were included in the validation dataset by WGS, and all of those were the Omicron VOC. The detection failure of the three targets falsely assigned 18 specimens to the presumptive Delta VOC, but WGS analysis recognized those as the Omicron VOC sublineage BA.1 and its sublineages (Table 1). A reverse situation was presented by two specimens, misclassified as the presumptive Omicron VOC but classified as the Delta VOC after WGS (Table 1). All 21 mislabeled specimens were submitted to the laboratory on January 7, 20, and 21 and tested within seven separate batches of the multiplexed RT-qPCR test. The *RdRp* Ct values of these 18 specimens with RT-qPCR target failures ranged from 11.91 to 36.32 with a median value of 24.85, and the Ct of the respective internal controls ranged from 23.49 to 28.60 with a median value of 26.06. Nevertheless, the test failed to detect ΔH69/V70, E484A, and N501Y.

The dominant quantity of the concordant results is illustrated by the correct classification rate and sensitivity exceeding 97% for each of the targets (Table 2), but the low prevalence of the Delta VOC (0.41%; 4/964; Figure 1) within the respective timeframe is pronounced. Thus, despite the positive prediction power exceeding 99% for all individual targets, the dataset composition derails the specificity measure for E484A and, especially, N501Y, while only two cases within the dataset (*n* = 964) were detected as false positives (Table 2). And, on the contrary, since the sequences with or without the presence of ΔH69/V70 were pronounced in reasonable proportions within the given timeframe and, consequently, the dataset, the obtained specificity measure agrees with the definition of the ΔH69/V70 target as a signature to distinguish between the BA.1 and BA.2 sublineages (Table 2).

## 4. Discussion

After a short co-circulation period with the Delta VOC, the Omicron VOC trod the path to achieve dominance at the end of 2021. Since then, the Omicron VOC has evolved into sublineages, assigned with varying alert levels by the World Health Organization (WHO [36]). The globally dominating sublineages as of September 2023 include variants of interest (XBB.1.5, XBB.1.16, and EG.5) and variants under monitoring (XBB.1.9.1 and XBB.1.9.2) [37]. In January 2022, the U.S. Food and Drug Administration released a statement limiting the use of two monoclonal antibody treatments [25]. Nevertheless, today, the multiple descendants of the BA.2 sublineage [38] have already exhausted the arsenal of all of the EU-approved monoclonal antibodies [39]. In 2021, the American Society for Microbiology issued a consensus review document outlining the clinical use of SARS-CoV-2 genotyping [9], but with the loss of susceptibility to monoclonal antibodies [40,41], the clinical scope of SARS-CoV-2 genotyping may eventually lie within the field of antiviral therapy [9,39].

The transition from Delta to the Omicron VOC with extensive immune evasion properties underlined the clinical necessity to introduce faster SARS-CoV-2 genotyping methods compared to WGS. For certain medical conditions, the need to start monoclonal antibody therapy right after the diagnosis of COVID-19 is crucial [9]. Therefore, therapeutic implications required a careful selection of targeted mutations to differentiate the Delta VOC and the Omicron VOC. The multiplexed RT-qPCR assay for the Omicron VOC employed in this study allowed for this differentiation. Moreover, since the BA.2 sublineage regained H69/V70, this assay enabled the simultaneous discrimination of the two presumptive sublineages, BA.1 and BA.2, and the pre-monitoring of their co-circulation, as well as the gradual increase in and the establishment of the latter variant. The H69/V70 within the BA.2 sublineage shielded it from detection by the *S* gene target failure assays based on this deletion only [11]. The two substitutions of the S protein targeted in this study, E484A and N501Y, have established a persistent niche in the variant landscape, are still present in the dominating sublineages of the Omicron VOC globally [42], and can still be used to recognize this variant. The process of targeting mutations residing outside the RBD of the S protein has been described to discriminate between sublineages BA.1 and BA.2, namely, ΔL24/P25/P26 and ins214EPE, with the addition of ΔE156/F157 to exclude the Delta VOC [20]. The BA.2 sublineage signature ΔL24/P25/P26 had been present at a low cumulative prevalence globally and had departed the multitude of the Omicron sublineages by the end of May 2023 [43]. The BA.1 signature ins214EPE was last detected at the end of March 2022 in Latvia (NMRL data). Discrimination of the Omicron VOC sublineages (BA.1, BA.2, and the subsequent BA.3, BA.4, and BA.5A) could be achieved by a set of several targets from the S protein, including ΔH69/V70, ins214EPE, and ΔG142/V143Y144, as well as 371F/373P/375F in the RBD [44]. The S protein and particularly the RBD, being the site of dense clustering of lineage-defining mutations, essentially determined the choice of the targets for the rapid genotyping of SARS-CoV-2. Several assays were aimed at other parts of its genome outside the hot spot of the selective pressure. For example, D3L from the N protein was grouped with ΔH69/V70 and N501Y to screen for the Alpha VOC [45]. A common mutation of the Alpha, Beta, and Gamma VOCs in *ORF1a* Δ3675–3677 was identified and chosen for the identification of these VOCs combined with ΔH69/V70 to differentiate the Alpha variant [19]. A deletion of ΔE31/R32/S33 in the N protein has been targeted to detect the Omicron VOC [46]. This signature has been sustained across all Omicron VOC lineages with a high cumulative prevalence and is still present worldwide [47] and in Latvia (NMRL data).

Since the declaration of COVID-19 as a global pandemic, the NMRL has executed the diagnostic response, monitoring, and genomic surveillance of SARS-CoV-2 and its variants. During the peak of the respiratory specimen inflow (Figure 1), the allele-specific RT-qPCR assay made it possible to genotype a higher number of SARS-CoV-2-positive specimens than the criteria for the WGS approach would allow, since those with a lower count of viral RNA copies and higher cycle threshold values could be included. Although WGS analysis requires considerable bioinformatics data processing and is resource intensive, the capacity of the hospital laboratory and NMRL enabled to include all SARS-CoV-2-positive specimens matching the criteria for WGS without the need to focus on special groups of patients, areas, clusters, or diagnostic failures of the rapid genotyping assays. This approach enabled the study described here and can provide for subsequent studies involving epidemiological and clinical data. SARS-CoV-2 VOC analysis by WGS revealed the diversity of the Omicron VOC PANGO sublineages (Table S1), beyond the presumptive VOC and lineages assigned by the faster genotyping approach. During the study period described, the sequencing efforts continued to adopt the all-inclusive approach, although it is admitted that the focus can be shifted to specific groups or clusters if the current circulating variant is fully dominating at >99% [34,48]. The Omicron VOC sublineage BA.2 was first confirmed by WGS in Latvia from a respiratory specimen collected on December 28. Both approaches documented the shift from BA.1 to the more advantageous [49] BA.2 sublineage. The transfer of the WGS results to informed decisions in the clinical and public health domains can still be hindered by comparatively longer turnaround times than faster genotyping methods relying on a few targets. The WGS analysis in the scope of this study served for validation of the RT-qPCR assay, and the results were not implemented into the test report to the clinician. We demonstrate that the sensitivity of the multiplexed RT-qPCR assay exceeded 96% for each of the signature mutations targeted by the test. The epidemiological scenery during the study period determined the specificity for the target N501Y, in particular. Confirmation by WGS showed that in 21 cases, the WHO label was assigned incorrectly by a multiplexed RT-qPCR test. The limitation of this study was the lack of purposive intra-assay precision tests run in triplicate and inter-assay tests run on separate days by RT-qPCR for the specimens with misassigned VOCs. Our study cannot be considered a preplanned validation study since the dataset represents the hospital laboratory setting within a very specific timeframe of the pandemic.

Several other studies have validated the performance of multiplexed RT-qPCR tests targeting some of the SARS-CoV-2 VOC signature mutations against the WGS or Sanger approach during the establishment and circulation of the currently de-escalated VOCs Alpha, Beta, Gamma, and Delta [13,14,16,50]. One of the studies reported 1.1% discordance by lineage (39/3551) [13], while another observed 100% concordance of 229 specimens by targets L452R, E484K, and N501Y [50]. Two commercial assays targeting L452R of the Delta VOC performed at 100% (67/67) and 94% accuracy (63/67) [16]. Targeting only one Omicron VOC-specific mutation, N501Y, yielded full concordance by lineage (29/30), with one discordant arising from a low viral load sample [48]. A set of targets consisting of two in the *ORF1ab* region, A2710T and T13195C, and one from the *S* gene, T547K, grouped with one Delta VOC-specific substitution in the S protein, T19R, correctly classified 99.8% of the 8870 Omicron samples during the transition period from Delta to Omicron in the USA [5]. Another set of 14 targets produced 100% concordance with the WGS of nine sequenced SARS-CoV-2 genomes ranging from the Beta to the Iota variants [18].

## 5. Conclusions

We conclude that the increased diagnostic capacity of the RT-qPCR genotyping approach enabled us to pre-monitor the pandemic scene in the central and regional hospitals of Latvia with acceptable accuracy.

## Figures and Tables

**Table 1:** The presumptive and actual WHO labels and PANGO lineages of the specimens with discordant target detections by multiplexed RT-qPCR, validated against the whole-genome sequencing (WGS) results.

*n*	Detection of Targets, Multiplexed RT-qPCR		Presence of Targets, WGS		*n*
2	ΔH69/V70, E484A, N501Y	Omicron, BA.1	-	Delta, B.1.617.2 Delta, AY.121	1 1
5	E484A, N501Y	Omicron, BA.2	ΔH69/V70, E484A, N501Y ΔH69/V70, E484A, N501Y ΔH69/V70, E484A, N501Y	Omicron, BA.1.17.2 Omicron, BA.1.1 Omicron, BA.2 *	1 2 2
1	ΔH69/V70, N501Y	Other	ΔH69/V70, E484A, N501Y	Omicron, BA.1.17.2	1
18	-	Delta	ΔH69/V70, E484A, N501Y ΔH69/V70, E484A, N501Y ΔH69/V70, E484A, N501Y ΔH69/V70, E484A, N501Y ΔH69/V70, E484A, N501Y ΔH69/V70, E484A, N501Y ΔH69/V70, E484A, N501Y	Omicron, BA.1 Omicron, BA.1.1 Omicron, BA.1.10 Omicron, BA.1.15 Omicron, BA.1.15.1 Omicron, BA.1.17 Omicron, BA.1.17.2	6 4 1 1 1 1 4

* The BA.2 lineage was assigned by PANGOLIN, despite the presence of ΔH69/V70.

**Table 2:** Detection accuracy and quality measures for recognition of ΔH69/V70, E484A, and N501Y by multiplexed RT-qPCR, validated against the WGS results.

Measures	Target		
	ΔH69/V70	E484A	N501Y
True positives (TP)	686	933	942
True negatives (TN)	255	10	2
False positives (FP)	21	19	18
False negatives (FN)	2	2	2
Correct classification rate (CCR), % (95% CI)	97.6 (96.7–98.6)	97.8 (96.9–98.7)	97.9 (97.0–98.8)
Sensitivity, % (95% CI)	97.0 (96.0–98.1)	98.0 (97.1–98.9)	98.1 (97.3–99.0)
Specificity, % (95% CI)	99.2 (98.7–99.8)	83.3 (81.0–85.7)	50.0 (46.8–53.2)
Positive predictive value (PPV), % (95% CI)	99.7 (99.4–100)	99.8 (99.5–100)	99.8 (99.5–100)
Negative predictive value (NPV), % (95% CI)	92.4 (90.7–94.1)	34.5 (31.5–37.5)	10.0 (8.1–11.9)

## Data Availability

SARS-CoV-2 genomes included in this study were uploaded to the GISAID (Global Initiative on Sharing All Influenza Data) database (https://gisaid.org/ accessed on 10 October 2023); the GISAID ID numbers for SARS-CoV-2 genomes are summarized in Supplementary File S1.

## Supplementary Materials

The following supporting information can be downloaded at: https://www.mdpi.com/article/10.3390/diagnostics13223467/s1. File S1: GISAID ID numbers for the SARS-CoV-2 genomes included in this study; Table S1: The presumptive and actual WHO labels and PANGO lineages of the specimens with discordant target detections by multiplexed RT-qPCR, validated against the WGS results.
