# Supplementary material for: Multiplexed RT-qPCR Coupled with Whole-Genome Sequencing to Monitor a SARS-CoV-2 Omicron Variant of Concern in a Hospital Laboratory Setting in Latvia
Source: Diagnostics (Basel). 2023 Nov 17;13(22):3467. doi: 10.3390/diagnostics13223467 (PMC10670528; doi:10.3390/diagnostics13223467)
Supplement: Supplementary file 1 [file diagnostics-13-03467-s001.zip › Supplementary File S1.pdf]

EPI_ISL_18367261, EPI_ISL_18367267, EPI_ISL_18367268, EPI_ISL_18367270, EPI_ISL_18367274, EPI_ISL_18367278, EPI_ISL_18367279, EPI_ISL_18367277, EPI_ISL_18367272, EPI_ISL_18367273, EPI_ISL_18367191, EPI_ISL_18367195, EPI_ISL_18367194, EPI_ISL_18367198, EPI_ISL_18367197, EPI_ISL_18367193, EPI_ISL_18367186, EPI_ISL_18367192, EPI_ISL_18367196, EPI_ISL_18367201, EPI_ISL_18367200, EPI_ISL_18367199, EPI_ISL_18367204, EPI_ISL_18367203, EPI_ISL_18367206, EPI_ISL_18367207, EPI_ISL_18367209, EPI_ISL_18367205, EPI_ISL_18367202, EPI_ISL_18367208, EPI_ISL_18367210, EPI_ISL_18367212, EPI_ISL_18367215, EPI_ISL_18367217, EPI_ISL_18367216, EPI_ISL_18367214, EPI_ISL_18367219, EPI_ISL_18367220, EPI_ISL_18367221, EPI_ISL_18367222, EPI_ISL_18367225, EPI_ISL_18367226, EPI_ISL_18367223, EPI_ISL_18367224, EPI_ISL_18367227, EPI_ISL_18367236, EPI_ISL_18367228, EPI_ISL_18367229, EPI_ISL_18367230, EPI_ISL_18367231, EPI_ISL_18367232, EPI_ISL_18367237, EPI_ISL_18367213, EPI_ISL_18367218, EPI_ISL_18367235, EPI_ISL_18367238, EPI_ISL_18367240, EPI_ISL_18367243, EPI_ISL_18367244, EPI_ISL_18367233, EPI_ISL_18367234, EPI_ISL_18367245, EPI_ISL_18367239, EPI_ISL_18367241, EPI_ISL_18367242, EPI_ISL_18367246, EPI_ISL_18367247, EPI_ISL_18367248, EPI_ISL_18367250, EPI_ISL_18367255, EPI_ISL_18367257, EPI_ISL_18367258, EPI_ISL_18367249, EPI_ISL_18367251, EPI_ISL_18367252, EPI_ISL_18367253, EPI_ISL_18367254, EPI_ISL_18367259, EPI_ISL_18367260, EPI_ISL_18367262, EPI_ISL_18367263, EPI_ISL_18367265, EPI_ISL_18367264, EPI_ISL_18367269, EPI_ISL_18367271, EPI_ISL_18367280, EPI_ISL_18367276, EPI_ISL_18367288, EPI_ISL_18367289, EPI_ISL_18367292, EPI_ISL_18367290, EPI_ISL_18367287, EPI_ISL_18367285, EPI_ISL_18367286, EPI_ISL_18367294, EPI_ISL_18367295, EPI_ISL_18367296, EPI_ISL_18367293, EPI_ISL_18367297, EPI_ISL_18367298, EPI_ISL_18367299, EPI_ISL_18367300, EPI_ISL_18367302, EPI_ISL_18367304, EPI_ISL_18367306, EPI_ISL_18367303, EPI_ISL_18367305, EPI_ISL_18367307, EPI_ISL_18367308, EPI_ISL_18367310, EPI_ISL_18367311, EPI_ISL_18367314, EPI_ISL_18367313, EPI_ISL_18367312, EPI_ISL_18367315, EPI_ISL_18367317, EPI_ISL_18367319, EPI_ISL_18367316, EPI_ISL_18367320, EPI_ISL_18367321, EPI_ISL_18367323, EPI_ISL_18367325, EPI_ISL_18367322, EPI_ISL_18367328, EPI_ISL_18367329, EPI_ISL_18367330, EPI_ISL_18367324, EPI_ISL_18367326, EPI_ISL_18367331, EPI_ISL_18367332, EPI_ISL_18367333, EPI_ISL_18367334, EPI_ISL_18367336, EPI_ISL_18367337, EPI_ISL_18367338, EPI_ISL_18367339, EPI_ISL_18367341, EPI_ISL_18367340, EPI_ISL_18367342, EPI_ISL_18367344, EPI_ISL_18367343, EPI_ISL_18367345, EPI_ISL_18367346, EPI_ISL_18367348, EPI_ISL_18367349, EPI_ISL_18367350, EPI_ISL_18367352, EPI_ISL_18367353, EPI_ISL_18367354, EPI_ISL_18367351, EPI_ISL_18367357, EPI_ISL_18367358, EPI_ISL_18367356, EPI_ISL_18367360, EPI_ISL_18367359, EPI_ISL_18367361, EPI_ISL_18367362, EPI_ISL_18367364, EPI_ISL_18367365, EPI_ISL_18367366, EPI_ISL_18367282, EPI_ISL_18367281, EPI_ISL_18367284, EPI_ISL_18367187, EPI_ISL_18367188, EPI_ISL_18367189, EPI_ISL_18367190, EPI_ISL_18367185, EPI_ISL_18367211, EPI_ISL_9705724, EPI_ISL_9705722, EPI_ISL_9705721, EPI_ISL_9705712, EPI_ISL_9705711, EPI_ISL_9705708, EPI_ISL_9705706, EPI_ISL_9705705, EPI_ISL_9705702, EPI_ISL_9705698, EPI_ISL_9705697, EPI_ISL_9705696, EPI_ISL_9705689, EPI_ISL_9705688, EPI_ISL_9705687,

EPI_ISL_9705685, EPI_ISL_9705684, EPI_ISL_9705683, EPI_ISL_9705682, EPI_ISL_9705681, EPI_ISL_9705679, EPI_ISL_9705678, EPI_ISL_9705677, EPI_ISL_9705676, EPI_ISL_9705675, EPI_ISL_9705674, EPI_ISL_9705673, EPI_ISL_9705671, EPI_ISL_9705668, EPI_ISL_9705664, EPI_ISL_9705631, EPI_ISL_9705630, EPI_ISL_9705629, EPI_ISL_9705628, EPI_ISL_9705626, EPI_ISL_9705625, EPI_ISL_9705623, EPI_ISL_9705620, EPI_ISL_9705619, EPI_ISL_9705616, EPI_ISL_9705613, EPI_ISL_9705612, EPI_ISL_9705606, EPI_ISL_9705604, EPI_ISL_9705601, EPI_ISL_9705599, EPI_ISL_9705598, EPI_ISL_9705597, EPI_ISL_9705594, EPI_ISL_9705593, EPI_ISL_9705590, EPI_ISL_9705589, EPI_ISL_9705584, EPI_ISL_9705582, EPI_ISL_9705579, EPI_ISL_9705577, EPI_ISL_9705575, EPI_ISL_9705574, EPI_ISL_9705572, EPI_ISL_9705571, EPI_ISL_9705569, EPI_ISL_9705568, EPI_ISL_9705567, EPI_ISL_9705566, EPI_ISL_9705556, EPI_ISL_9705555, EPI_ISL_9705554, EPI_ISL_9705553, EPI_ISL_9705550, EPI_ISL_9705549, EPI_ISL_9705546, EPI_ISL_9705545, EPI_ISL_9705544, EPI_ISL_9705538, EPI_ISL_9705532, EPI_ISL_9705529, EPI_ISL_9705528, EPI_ISL_9705526, EPI_ISL_9705524, EPI_ISL_9705522, EPI_ISL_9705521, EPI_ISL_9705520, EPI_ISL_9705519, EPI_ISL_9705518, EPI_ISL_9705513, EPI_ISL_9705512, EPI_ISL_9705508, EPI_ISL_9705506, EPI_ISL_9705503, EPI_ISL_9705502, EPI_ISL_9705496, EPI_ISL_9705494, EPI_ISL_9705490, EPI_ISL_9705489, EPI_ISL_9705486, EPI_ISL_9705484, EPI_ISL_9705483, EPI_ISL_9705482, EPI_ISL_9705481, EPI_ISL_9705480, EPI_ISL_9705473, EPI_ISL_9705472, EPI_ISL_9705465, EPI_ISL_9705464, EPI_ISL_9705462, EPI_ISL_9705457, EPI_ISL_9705453, EPI_ISL_9705452, EPI_ISL_9705450, EPI_ISL_9705444, EPI_ISL_9705443, EPI_ISL_9705442, EPI_ISL_9705440, EPI_ISL_9705435, EPI_ISL_9705428, EPI_ISL_9705427, EPI_ISL_9705425, EPI_ISL_9705422, EPI_ISL_9705419, EPI_ISL_9705417, EPI_ISL_9705413, EPI_ISL_9705376, EPI_ISL_9705375, EPI_ISL_9705373, EPI_ISL_9705371, EPI_ISL_9705361, EPI_ISL_9705360, EPI_ISL_9705355, EPI_ISL_9705352, EPI_ISL_9705350, EPI_ISL_9705344, EPI_ISL_9705343, EPI_ISL_13631122, EPI_ISL_13631060, EPI_ISL_13630303, EPI_ISL_13630300, EPI_ISL_13630288, EPI_ISL_13630286, EPI_ISL_13630273, EPI_ISL_13630245, EPI_ISL_13630229, EPI_ISL_13630225, EPI_ISL_13630218, EPI_ISL_13630215, EPI_ISL_13630213, EPI_ISL_13630212, EPI_ISL_13630209, EPI_ISL_13630205, EPI_ISL_13630200, EPI_ISL_13630186, EPI_ISL_13630185, EPI_ISL_13630184, EPI_ISL_13630183, EPI_ISL_13630174, EPI_ISL_13630168, EPI_ISL_13630167, EPI_ISL_13630163, EPI_ISL_13630161, EPI_ISL_13630159, EPI_ISL_13630149, EPI_ISL_13630145, EPI_ISL_13630144, EPI_ISL_13630138, EPI_ISL_13630131, EPI_ISL_13630130, EPI_ISL_13630125, EPI_ISL_13630120, EPI_ISL_13630117, EPI_ISL_13630116, EPI_ISL_13630115, EPI_ISL_13630110, EPI_ISL_13630108, EPI_ISL_13630107, EPI_ISL_13630101, EPI_ISL_13630099, EPI_ISL_13630089, EPI_ISL_13630088, EPI_ISL_13630074, EPI_ISL_13630067, EPI_ISL_13630065, EPI_ISL_13630064, EPI_ISL_13630061, EPI_ISL_13630055, EPI_ISL_13630043, EPI_ISL_13630039, EPI_ISL_13630038, EPI_ISL_13630037, EPI_ISL_13630030, EPI_ISL_13630027, EPI_ISL_13630023, EPI_ISL_13630021, EPI_ISL_13630019, EPI_ISL_13630015, EPI_ISL_13630014, EPI_ISL_13630011, EPI_ISL_13630009, EPI_ISL_13630008, EPI_ISL_13630007, EPI_ISL_13629996,

EPI_ISL_13629989, EPI_ISL_13629986, EPI_ISL_13629984, EPI_ISL_13629981, EPI_ISL_13629979, EPI_ISL_13629977, EPI_ISL_13629975, EPI_ISL_13629973, EPI_ISL_13629970, EPI_ISL_13629960, EPI_ISL_13629954, EPI_ISL_13629953, EPI_ISL_13629951, EPI_ISL_13629949, EPI_ISL_13629940, EPI_ISL_13629935, EPI_ISL_13629926, EPI_ISL_13629922, EPI_ISL_13629913, EPI_ISL_13629912, EPI_ISL_13629910, EPI_ISL_13629909, EPI_ISL_13629908, EPI_ISL_13629907, EPI_ISL_13629904, EPI_ISL_13629901, EPI_ISL_13629900, EPI_ISL_13629899, EPI_ISL_13629896, EPI_ISL_13629887, EPI_ISL_13629880, EPI_ISL_13629879, EPI_ISL_13629878, EPI_ISL_13629876, EPI_ISL_13629875, EPI_ISL_13629874, EPI_ISL_13629870, EPI_ISL_13629868, EPI_ISL_13629867, EPI_ISL_13629865, EPI_ISL_13629856, EPI_ISL_13629852, EPI_ISL_13629850, EPI_ISL_13629849, EPI_ISL_13629844, EPI_ISL_13629842, EPI_ISL_13629838, EPI_ISL_13629830, EPI_ISL_13629823, EPI_ISL_13629817, EPI_ISL_13629808, EPI_ISL_13629795, EPI_ISL_13629794, EPI_ISL_13629791, EPI_ISL_13629789, EPI_ISL_13629788, EPI_ISL_13629783, EPI_ISL_13629780, EPI_ISL_13629776, EPI_ISL_13629772, EPI_ISL_13629770, EPI_ISL_13629764, EPI_ISL_13629758, EPI_ISL_13629756, EPI_ISL_13629755, EPI_ISL_13629752, EPI_ISL_13629750, EPI_ISL_13629747, EPI_ISL_13629744, EPI_ISL_13629742, EPI_ISL_13629735, EPI_ISL_13629726, EPI_ISL_13629725, EPI_ISL_13629723, EPI_ISL_13629721, EPI_ISL_13629716, EPI_ISL_13629715, EPI_ISL_13629712, EPI_ISL_13629711, EPI_ISL_13629706, EPI_ISL_13629699, EPI_ISL_13629694, EPI_ISL_13629693, EPI_ISL_13629692, EPI_ISL_13629691, EPI_ISL_13629690, EPI_ISL_13629687, EPI_ISL_13629677, EPI_ISL_13629676, EPI_ISL_13629667, EPI_ISL_13629664, EPI_ISL_13629660, EPI_ISL_13629659, EPI_ISL_13629654, EPI_ISL_13629649, EPI_ISL_13629637, EPI_ISL_13629631, EPI_ISL_13629626, EPI_ISL_13629624, EPI_ISL_13629618, EPI_ISL_13629613, EPI_ISL_13629608, EPI_ISL_13629607, EPI_ISL_13629604, EPI_ISL_13629601, EPI_ISL_13629592, EPI_ISL_13629579, EPI_ISL_13629577, EPI_ISL_13629576, EPI_ISL_13629571, EPI_ISL_13629569, EPI_ISL_13629566, EPI_ISL_13629564, EPI_ISL_13629558, EPI_ISL_13629554, EPI_ISL_13629553, EPI_ISL_13629544, EPI_ISL_13629540, EPI_ISL_13629537, EPI_ISL_13629532, EPI_ISL_13629531, EPI_ISL_13629530, EPI_ISL_13629529, EPI_ISL_13629527, EPI_ISL_13629524, EPI_ISL_13629523, EPI_ISL_13629521, EPI_ISL_13629510, EPI_ISL_13629502, EPI_ISL_13629499, EPI_ISL_13629498, EPI_ISL_13629497, EPI_ISL_13629496, EPI_ISL_13629495, EPI_ISL_13629489, EPI_ISL_13629488, EPI_ISL_13629476, EPI_ISL_13629472, EPI_ISL_13629470, EPI_ISL_13629469, EPI_ISL_13629465, EPI_ISL_13629460, EPI_ISL_13629454, EPI_ISL_13629451, EPI_ISL_13629447, EPI_ISL_13629436, EPI_ISL_13629431, EPI_ISL_13629421, EPI_ISL_13629404, EPI_ISL_13629400, EPI_ISL_13629396, EPI_ISL_13629392, EPI_ISL_13629389, EPI_ISL_13629387, EPI_ISL_13629386, EPI_ISL_13629384, EPI_ISL_13629383, EPI_ISL_13629377, EPI_ISL_13629376, EPI_ISL_13629367, EPI_ISL_13629366, EPI_ISL_13629364, EPI_ISL_13629362, EPI_ISL_13629359, EPI_ISL_13629356, EPI_ISL_13629354, EPI_ISL_13629353, EPI_ISL_13629350, EPI_ISL_13629348, EPI_ISL_13629329, EPI_ISL_13629326, EPI_ISL_13629325, EPI_ISL_13629320, EPI_ISL_13629318, EPI_ISL_13629307, EPI_ISL_13629306, EPI_ISL_13629305, EPI_ISL_13629300, EPI_ISL_13629294, EPI_ISL_13629287, EPI_ISL_13629264, EPI_ISL_13629259, EPI_ISL_13629253, EPI_ISL_13629244,

EPI_ISL_13629240, EPI_ISL_13629237, EPI_ISL_13629236, EPI_ISL_13629235, EPI_ISL_13629233, EPI_ISL_13629230, EPI_ISL_13629229, EPI_ISL_13629223, EPI_ISL_13629221, EPI_ISL_13629208, EPI_ISL_13629204, EPI_ISL_13629186, EPI_ISL_13629183, EPI_ISL_13629179, EPI_ISL_13629178, EPI_ISL_13629173, EPI_ISL_13629169, EPI_ISL_13629168, EPI_ISL_13629167, EPI_ISL_13629166, EPI_ISL_13629165, EPI_ISL_13629156, EPI_ISL_13629152, EPI_ISL_13629147, EPI_ISL_13629146, EPI_ISL_13629144, EPI_ISL_13629140, EPI_ISL_13629131, EPI_ISL_13629130, EPI_ISL_13629127, EPI_ISL_13629114, EPI_ISL_13629113, EPI_ISL_13629100, EPI_ISL_13629095, EPI_ISL_13629063, EPI_ISL_13629053, EPI_ISL_13629044, EPI_ISL_13629041, EPI_ISL_13629035, EPI_ISL_13629027, EPI_ISL_13629023, EPI_ISL_13629015, EPI_ISL_13629014, EPI_ISL_13628993, EPI_ISL_13628992, EPI_ISL_13628980, EPI_ISL_13628979, EPI_ISL_13628973, EPI_ISL_13628967, EPI_ISL_13628960, EPI_ISL_13628959, EPI_ISL_13628953, EPI_ISL_13628952, EPI_ISL_13628947, EPI_ISL_13628944, EPI_ISL_13628942, EPI_ISL_13628939, EPI_ISL_13628926, EPI_ISL_13628922, EPI_ISL_13628920, EPI_ISL_13628917, EPI_ISL_13628907, EPI_ISL_13628902, EPI_ISL_13628889, EPI_ISL_13628886, EPI_ISL_13628875, EPI_ISL_13628873, EPI_ISL_13628870, EPI_ISL_13628864, EPI_ISL_13628862, EPI_ISL_13628860, EPI_ISL_13628858, EPI_ISL_13628851, EPI_ISL_13628850, EPI_ISL_13628847, EPI_ISL_13628838, EPI_ISL_13628837, EPI_ISL_13628831, EPI_ISL_13628830, EPI_ISL_13628817, EPI_ISL_13628813, EPI_ISL_13628811, EPI_ISL_13628809, EPI_ISL_13628797, EPI_ISL_13628795, EPI_ISL_13628792, EPI_ISL_13628783, EPI_ISL_13628781, EPI_ISL_13628779, EPI_ISL_13628772, EPI_ISL_13628767, EPI_ISL_13628766, EPI_ISL_13628756, EPI_ISL_13628743, EPI_ISL_13628742, EPI_ISL_13628730, EPI_ISL_13628729, EPI_ISL_13628728, EPI_ISL_13628725, EPI_ISL_13628716, EPI_ISL_13628712, EPI_ISL_13628705, EPI_ISL_13628704, EPI_ISL_13628703, EPI_ISL_13628693, EPI_ISL_13628688, EPI_ISL_13628681, EPI_ISL_13628680, EPI_ISL_13628668, EPI_ISL_13628667, EPI_ISL_13628655, EPI_ISL_13628651, EPI_ISL_13628649, EPI_ISL_13628648, EPI_ISL_13628638, EPI_ISL_13628635, EPI_ISL_13628630, EPI_ISL_13628628, EPI_ISL_13628624, EPI_ISL_13628621, EPI_ISL_13628617, EPI_ISL_13628616, EPI_ISL_13628613, EPI_ISL_13628598, EPI_ISL_13628597, EPI_ISL_13628592, EPI_ISL_13628584, EPI_ISL_13628582, EPI_ISL_13628577, EPI_ISL_13628574, EPI_ISL_13628571, EPI_ISL_13628569, EPI_ISL_13628565, EPI_ISL_13628550, EPI_ISL_13628542, EPI_ISL_13628537, EPI_ISL_13628533, EPI_ISL_13628530, EPI_ISL_13628522, EPI_ISL_13628512, EPI_ISL_13628505, EPI_ISL_13628504, EPI_ISL_13628497, EPI_ISL_13628496, EPI_ISL_13628490, EPI_ISL_13628487, EPI_ISL_13628482, EPI_ISL_13628475, EPI_ISL_13628460, EPI_ISL_13628448, EPI_ISL_13628444, EPI_ISL_13628431, EPI_ISL_13628427, EPI_ISL_13628421, EPI_ISL_13628408, EPI_ISL_13628396, EPI_ISL_13628394, EPI_ISL_13628393, EPI_ISL_13628392, EPI_ISL_13628385, EPI_ISL_13628384, EPI_ISL_13628364, EPI_ISL_13628361, EPI_ISL_13628356, EPI_ISL_13628354, EPI_ISL_13628348, EPI_ISL_13628344, EPI_ISL_13628329, EPI_ISL_13628327, EPI_ISL_13628324, EPI_ISL_13628316, EPI_ISL_13628314, EPI_ISL_13628310, EPI_ISL_13628307, EPI_ISL_13628304, EPI_ISL_13628294, EPI_ISL_13628293, EPI_ISL_13628291, EPI_ISL_13628290, EPI_ISL_13628284, EPI_ISL_13628283, EPI_ISL_13628282, EPI_ISL_13628278, EPI_ISL_13628275,

EPI_ISL_13628270, EPI_ISL_13628244, EPI_ISL_13628228, EPI_ISL_13628226, EPI_ISL_13628225, EPI_ISL_13628223, EPI_ISL_13628220, EPI_ISL_13628214, EPI_ISL_13628211, EPI_ISL_13628206, EPI_ISL_13628202, EPI_ISL_13628200, EPI_ISL_13628193, EPI_ISL_13628190, EPI_ISL_13628176, EPI_ISL_13628155, EPI_ISL_13628150, EPI_ISL_13628142, EPI_ISL_13628130, EPI_ISL_13628129, EPI_ISL_13628127, EPI_ISL_13628112, EPI_ISL_13628110, EPI_ISL_13628100, EPI_ISL_13628092, EPI_ISL_13628091, EPI_ISL_13628089, EPI_ISL_13628087, EPI_ISL_13628085, EPI_ISL_13628076, EPI_ISL_13628071, EPI_ISL_13628067, EPI_ISL_13628055, EPI_ISL_13628044, EPI_ISL_13628041, EPI_ISL_13628034, EPI_ISL_13628029, EPI_ISL_13628025, EPI_ISL_13628024, EPI_ISL_13628021, EPI_ISL_13628008, EPI_ISL_13628005, EPI_ISL_13628002, EPI_ISL_13628001, EPI_ISL_13627998, EPI_ISL_13627990, EPI_ISL_13627986, EPI_ISL_13627969, EPI_ISL_13627956, EPI_ISL_13627955, EPI_ISL_13627951, EPI_ISL_13627948, EPI_ISL_13627941, EPI_ISL_13627937, EPI_ISL_13627934, EPI_ISL_13627924, EPI_ISL_13627921, EPI_ISL_13627918, EPI_ISL_13627915, EPI_ISL_13627910, EPI_ISL_13627906, EPI_ISL_13627904, EPI_ISL_13627895, EPI_ISL_13627893, EPI_ISL_13627891, EPI_ISL_13627886, EPI_ISL_13627881, EPI_ISL_13627873, EPI_ISL_13627870, EPI_ISL_13627863, EPI_ISL_13627855, EPI_ISL_13627853, EPI_ISL_13627845, EPI_ISL_13627842, EPI_ISL_13627839, EPI_ISL_13627838, EPI_ISL_13627837, EPI_ISL_13627826, EPI_ISL_13627817, EPI_ISL_13627815, EPI_ISL_13627814, EPI_ISL_13627803, EPI_ISL_13627799, EPI_ISL_13627790, EPI_ISL_13627786, EPI_ISL_13627780, EPI_ISL_13627779, EPI_ISL_13627776, EPI_ISL_13627774, EPI_ISL_13627771, EPI_ISL_13627770, EPI_ISL_13627760, EPI_ISL_13627759, EPI_ISL_13627758, EPI_ISL_13627752, EPI_ISL_13627751, EPI_ISL_13627748, EPI_ISL_13627730, EPI_ISL_13627727, EPI_ISL_13627723, EPI_ISL_13627716, EPI_ISL_13627709, EPI_ISL_13627702, EPI_ISL_13627700, EPI_ISL_13627686, EPI_ISL_13627679, EPI_ISL_13627672, EPI_ISL_13627659, EPI_ISL_13627655, EPI_ISL_13627643, EPI_ISL_13627639, EPI_ISL_13627626, EPI_ISL_13627622, EPI_ISL_13627621, EPI_ISL_13627620, EPI_ISL_13627611, EPI_ISL_13627593, EPI_ISL_13627592, EPI_ISL_13627587, EPI_ISL_13627582, EPI_ISL_13627576, EPI_ISL_13627572, EPI_ISL_13627571, EPI_ISL_13627568, EPI_ISL_13627567, EPI_ISL_13627565, EPI_ISL_13627560, EPI_ISL_13627553, EPI_ISL_13627548, EPI_ISL_13627517, EPI_ISL_13627512, EPI_ISL_13627499, EPI_ISL_13627483, EPI_ISL_13627479, EPI_ISL_13627477, EPI_ISL_13627475, EPI_ISL_13627467, EPI_ISL_13627465, EPI_ISL_13627456, EPI_ISL_13627438, EPI_ISL_13627427, EPI_ISL_13627422, EPI_ISL_13627419, EPI_ISL_13627409, EPI_ISL_13627403, EPI_ISL_13627385, EPI_ISL_13627381, EPI_ISL_13627376, EPI_ISL_13627363, EPI_ISL_13627354, EPI_ISL_13627346, EPI_ISL_13627328, EPI_ISL_13627296, EPI_ISL_13627295, EPI_ISL_13627283, EPI_ISL_13627277, EPI_ISL_13627273, EPI_ISL_13627264, EPI_ISL_13627259, EPI_ISL_13627258, EPI_ISL_13627245, EPI_ISL_13627238, EPI_ISL_13627216, EPI_ISL_13627202, EPI_ISL_13627196, EPI_ISL_13627191, EPI_ISL_13627178, EPI_ISL_13627177, EPI_ISL_13627176, EPI_ISL_13627167, EPI_ISL_13627166, EPI_ISL_13627163, EPI_ISL_13627162, EPI_ISL_13627157, EPI_ISL_13627152, EPI_ISL_13627151, EPI_ISL_13627133, EPI_ISL_13627125, EPI_ISL_13627109, EPI_ISL_13627105, EPI_ISL_13627094, EPI_ISL_13627085, EPI_ISL_13627084, EPI_ISL_13627081,

EPI_ISL_13627064, EPI_ISL_13627059, EPI_ISL_13627055, EPI_ISL_13627042, EPI_ISL_13627032, EPI_ISL_13627011, EPI_ISL_13627010, EPI_ISL_13627007, EPI_ISL_13627003, EPI_ISL_13626991, EPI_ISL_13626982, EPI_ISL_13626958, EPI_ISL_13626939, EPI_ISL_13626938, EPI_ISL_13626922, EPI_ISL_13626920, EPI_ISL_13626917, EPI_ISL_13626913, EPI_ISL_13626912, EPI_ISL_13626911, EPI_ISL_13626888, EPI_ISL_13626873, EPI_ISL_13626870, EPI_ISL_13626849, EPI_ISL_13626848, EPI_ISL_13626825, EPI_ISL_13626823, EPI_ISL_13626815, EPI_ISL_13626807, EPI_ISL_13626797, EPI_ISL_13626792, EPI_ISL_13626790, EPI_ISL_13626778, EPI_ISL_13626776, EPI_ISL_13626775, EPI_ISL_13626770, EPI_ISL_13626768, EPI_ISL_13626766, EPI_ISL_13626744, EPI_ISL_13626738, EPI_ISL_13626729, EPI_ISL_13626728, EPI_ISL_13626718, EPI_ISL_13626715.
