# Supplementary material for: Multiplexed RT-qPCR Coupled with Whole-Genome Sequencing to Monitor a SARS-CoV-2 Omicron Variant of Concern in a Hospital Laboratory Setting in Latvia
Source: Diagnostics (Basel). 2023 Nov 17;13(22):3467. doi: 10.3390/diagnostics13223467 (PMC10670528; doi:10.3390/diagnostics13223467)
Supplement: Supplementary file 1 [file diagnostics-13-03467-s001.zip › Supplementary Table S1.pdf]

Table S1.

The presumptive and actual WHO labels and PANGO lineages of the specimens with discordant target detections by multiplexed RT-qPCR, validated against WGS results

**Multiplexed RT-qPCR**							
ΔH69/V70, E484A, N501Y		E484A, N501Y		ΔH69/V70, N501Y		-	
BA.1	677	BA.2	251	Other	8	Delta	2
**WGS**							
BA.1	136	BA.2	204	BA.1.1	8	AY.4	1
BA.1.1	221	BA.2.1	3			B.1.617.2	1
BA.1.1.1	18	BA.2.22	2				
BA.1.1.11	2	BA.2.3	1				
BA.1.1.14	11	BA.2.9	41				
BA.1.1.15	2						
BA.1.10	1						
BA.1.13	3						
BA.1.14	1						
BA.1.15	76						
BA.1.15.1	14						
BA.1.16	5						
BA.1.17	38						
BA.1.17.2	104						
BA.1.18	13						
BA.1.20	9						
BA.1.21	18						
BD.1	5						
